# Emerging landscape of circFNDC3B and its role in human malignancies

**DOI:** 10.3389/fonc.2023.1097956

**Published:** 2023-01-31

**Authors:** Kai Sun, Huibao Yao, Peizhi Zhang, Yanning Sun, Jian Ma, Qinghua Xia

**Affiliations:** ^1^ Department of Urology, Shandong Province Hospital, Shandong University, Jinan, China; ^2^ Department of Urology, The Affiliated Yantai Yuhuangding Hospital of Qingdao University, Yantai, Shandong, China

**Keywords:** circRNAs, circFNDC3B, cancers, diseases, biomarker

## Abstract

In recent years, more attention has been paid to expanding the abundance of Circular RNAs (circRNAs), while the circRNAs that have been found to have significant functions have not been studied in different diseases. CircFNDC3B is one of the most researched circRNAs generated from fibronectin type III domain-containing protein 3B (FNDC3B) gene. Accumulating researches have reported the multiple functions of circFNDC3B in different cancer types and other non-neoplastic diseases, and predicted that circFNDC3B might be a potential biomarker. Notably, circFNDC3B can play roles in different diseases by binding to various microRNAs (miRNAs), binding to RNA-binding proteins (RBPs), or encoding functional peptides. This paper systematically summarizes the biogenesis and function of circRNAs, reviews and discusses the roles and molecular mechanisms of circFNDC3B and its target genes in different cancers and non-neoplastic diseases, which will do favor to broaden our comprehension of the function of circRNAs and facilitate subsequent research on circFNDC3B.

## Introduction

Following the extensive studies of long noncoding RNAs (lncRNAs) and microRNAs (miRNAs) in various diseases (1–3), circular RNAs (circRNAs), another important member of the non-coding RNAs (ncRNAs) family, have also been found to be widely involved in tumors and other diseases and have been exhibited to be valuable diagnostic and prognostic biomarkers (4–6).

**Figure 1:**
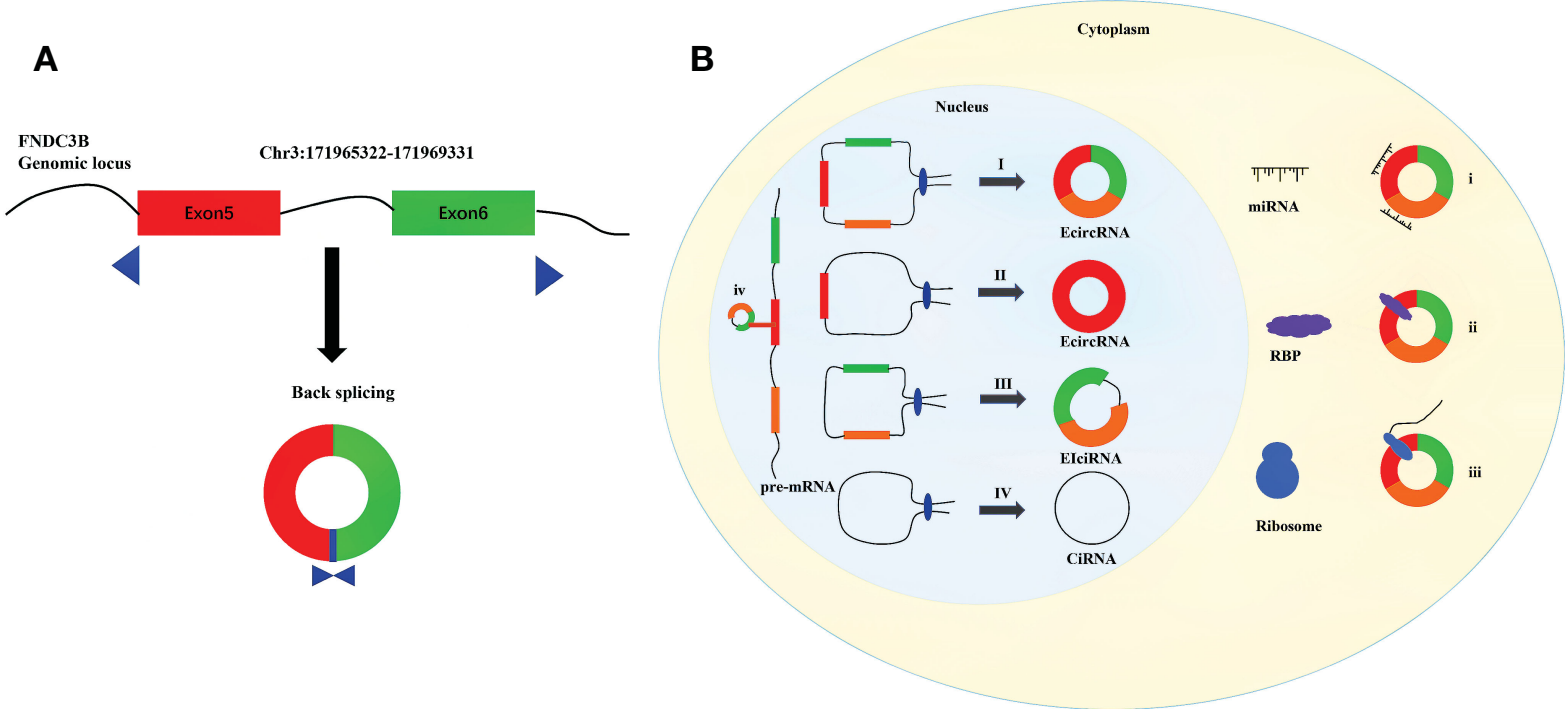
Diagram for biogenesis and functions of circRNAs. **(A)** Schematic diagram of the formation of circFNDC3B. **(B)** I. The downstream 5 ‘end of one exon is connected to the upstream 3’ end of another exon and cyclized to produce EcircRNA. II. The 5 ‘end of certain exon is attached to the 3’ end of the same exon to produce EcircRNA. III. Intron pairing driven circularization to produce EIciRNA. IV Conserved sequences near the spliceosome to produce CiRNA. і. MiRNA sponges. ii. Interaction with functional proteins. III. Translation into peptides and/or proteins. iv. Regulation of gene transcription.

CircRNAs are endogenous single-stranded ncRNAs produced by protein-coding genes (7, 8). First identified in viruses, circRNAs were initially considered to be by-products of selective splicing, and were supposed to have limited function (9–11). Benefiting from recent advances in transcriptome sequencing and bioinformatics, a large number of circRNAs have been identified (12–15). Unlike linear RNAs, a covalent closed-loop structure with the absence of 5’ cap and 3’ polyadenylated tail is the crucial feature of circRNAs (16–18). Compared with linear mRNAs, circRNAs are less susceptible to degradation by RNA exonuclease and RNase R because of their stable circular structure (14, 19). CircRNAs are generally expressed in various organisms, especially in distinct developmental stages and tissues (19–21). And circRNAs are found to be evolutionarily conserved across species (15). By the means of regulating the expression of parental genes, combining with RNA-binding proteins (RBPs), sponging miRNAs, and acting as templates for translation, circRNAs have been considered as significant regulatory factors at the levels of transcription and posttranscription (22–25). CircRNAs have been proved to possess significant biological functions in the development of human physiology and pathology (26–28). CircFNDC3B is generated from the back splicing of exons 5 and 6 of Fibronectin Type III Domain Containing 3B (FNDC3B), which is located on the human chromosome 3q26.31 (**Figure 1A**). Studies have reported that circFNDC3B is a highly stable circular RNA in the cytoplasm since the half-life of circFNDC3B exceeds 24 hours while that of linear FNDC3B was only 4 hours (29, 30). In addition, circFNDC3B is dysregulated in many tumors and participates in the process of proliferation, invasion, migration, apoptosis and epithelial-mesenchymal transition (EMT). More than that, circFNDC3B also plays an important role in non-tumor diseases such as myocardial infection and abnormal aortic aneurysm. The present review discusses the biogenesis and probable functions of circRNAs, and concentrates on recent advancements on the researches of circFNDC3B and its connections with cancers and other diseases. The objective of the study is to enhance the understanding on circFNDC3B and contribute to the researches of the regulatory roles of circFNDC3B in disease development and progression.

## The biogenesis of circRNAs

CircRNAs can be divided into three categories according to their sources: exon derived circRNAs (ecircRNAs or ecRNAs), intron derived circRNAs (ciRNAs), and circRNAs composed of exons and introns (EIciRNAs) (31–33).

The formation of circRNAs are different from the standard cutting mode of linear RNAs, which are cut by backsplicing (34). The existing circRNAs formation models are mainly composed of the following four kinds: 1) Exon skipping (35). In pre-mRNA, the combination of splicing donor and splicing receptor shortens the distance between the original unconnected exons and causes the exons skipping (36). By skipping exons, exons containing lariat are formed, which are generated according to the normal splicing mechanism (35). Then the lariat undergoes internal splicing to remove introns and produce a circular RNA composed of exons (ecRNAs) (8, 10). 2) Intron-Pairing-Driven Circularization. In this model, two complementary introns in pre-mRNA form a ring structure containing multiple exons and introns through base pairing (37, 38). Subsequently, the structure removes the intron to form ecRNAs or retains the intron to form EIciRNAs (10). 3) RBP-driven circularization. Some studies have shown that RBPs, such as FUS, QKI and MBL and so on, are involved in the formation of circRNAs (39–41). It binds to two non-adjacent introns in pre-mRNA and the introns were then removed or not removed to produce ecRNAs or EIciRNAs (42). 4) ciRNAs formation mode. Unlike the synthesis of EIciRNAs and ecRNAs, ciRNAs are composed of 2’-5’ phospholipid linked nucleotides (43, 44). The lariat formed after intron splicing forms a stable ciRNAs by combining with 5’ splice site rich in GU and branchpoint sitem rich in C (**Figure 1B**) (38).

## The function of circRNAs

CircRNAs is widely distributed in cells and can regulate various physiological processes in different ways. Circular RNAs with introns (EIciRNAs and ciRNAs) usually play a role in the nucleus (23). The circular RNAs without intron go out of the nucleus and into the cytoplasm, playing the role of miRNA sponge, RBP and protein translation (**Figure 1B**) (45).

### CircRNAs act as gene transcriptional regulators

EIciRNAs enhance parental gene transcription in a cis manner by interacting with U1 small nuclear ribonucleoproteins (snRNPs) and RNA Pol II (31). CiRNAs directly bind RNA Pol II, actively regulate the transcriptional activity of Pol II, so as to affect the transcription of parental genes (38). For example, EIciRNAs such as circPAIP2 and circEIF3J, ciRNAs such as circANKRD52 can combine with the RNA Pol II and form a complex, to regulate the gene transcription and expression (31, 38).

### CircRNAs act as miRNA sponges

MiRNAs are a kind of common non -coding RNAs with a length of about 21nt. They can directly bind to mRNA through base complementary pairing, thus inhibiting mRNA translation (46). As a member of competitive endogenous RNA (ceRNAs), circular RNA can inhibit the activity of miRNA in the form of competitive inhibition by adsorbing specific miRNA, so as to alleviate its inhibition effect on target genes (47–49). For example, ciRS-7 is a special type of circular RNA sponge of miR-7, with more than 70 miR-7 binding sites (50). ciRS-7 can affect the binding ability of miR-7 to target mRNA and participate in the occurrence and development of various cancers and neurodegenerative diseases (51, 52).

### CircRNAs act as RBP sponges

CircRNAs combine with RBPs to form RNA protein complexes (RPCs), which play an important role in physiological activities (47, 53). circMbl and MBL proteins are produced by the same pre-mRNA, circMbl binds MBL protein and prevents it from performing neural functions (39, 54). circANRIL is related to atherosclerotic cardiovascular disease. It inhibits the production of ribosomes in vascular smooth muscle cells and macrophages by binding to ribosomal 60S subunit, resulting in atherosclerosis related cell death (55).

### CircRNAs act as templates for protein translation

In previous studies, because circRNAs are classified as noncoding RNAs, their role in translation has never attracted attention. In fact, circular RNA contains an internal ribosomal entry site (IRESs) that can be translated effectively and has the ability to bind to open reading frames (ORFs), which proves that circular RNA has the function of encoding proteins or peptides (56–59). The circRNADb website records 72 circRNAs that can express proteins in humans. In addition, there are 250 circRNAs with translatable coding potential (60).

## The roles of circFNDC3B in cancers

### Bladder cancer

As one of the most frequent cancer worldwide, the molecular mechanisms underlying the development of bladder cancer (BCa) have been extensively studied by researchers (11, 61). CircFNDC3B has been considered to participant in the invasion and metastasis of BCa (62).

Liu et al. (62) established a cell invasion model to screen out the highly invasive cells and poorly invasive cells. The researchers used qRT-PCR to detect the expression of circRNAs and thus identify those associated with invasion. CircFNDC3B showed low expression levels both in bladder cancer tissues and in highly invasive T24 cells. A series of *in vivo* and *in vitro* experiments were conducted by Liu et al. They concluded that circFNDC3B could inhibit the proliferation, migration and invasion of BCa cells. *In vivo* experiments with BCa cell lines demonstrated that circFNDC3B might inhibit tumor growth and lymphatic metastasis. Bioinformatics analyses, pull-down assay employing a biotin-coupled circFNDC3B probe, luciferase reporter assays, biotinylated-miR-1178-3p RNA pull-down experiments, and double FISH assay were conducted in sequence. The results suggested that circFNDC3B could be the sponge of miR-1178-3p to inhibit its activity in BCa cells. Furthermore, miR-1178-3p was identified to target the 5′UTR of G3BP2, a member of the Ras-GTPase-activating protein (RasGAP) SH3 domain-binding protein (G3BP) family (63, 64). The downregulation of miR-1178-3p by circFNDC3B inhibited the level of G3BP2, which in turn suppressed tumor cell proliferation. All findings demonstrated that circFNDC3B can restrain the pathogenesis of BCa through the miR-1178-3p-G3BP2 axis. The results indicated a probable therapeutic target for BCa (62).

### Gastric cancer

Gastric cancer (GC) is considered to be one of the most severe tumors of digestive system, with high morbidity and mortality (65, 66). Recently, the potential mechanisms of circFNDC3B in GC pathogenesis have been researched (67, 68).

In a study by Hong et al., the expression of circFNDC3B was examined in both more invasive and migrative GC cell lines and less invasive and migrative GC cell lines (67). CircFNDC3B exhibited higher levels in GC cell lines with the worst malignant phenotype than that in other GC cell lines and normal GC cell lines. In GC cell lines, circFNDC3B was found to promote the invasion and migration of GC cells by enhancing the activity of EMT, which takes part in cancer metastasis (69). Subsequently, the combination of circFNDC3B and insulin‐like growth factor 2 binding protein 3 (IGF2BP3), an RNA binding protein (RBP) connected with varieties of malignant tumors (70), was detected by RIP assay, and the binding of IGF2BP3 and CD44 mRNA was identified by RNA pull down assay. The findings suggested that circFNDC3B mediates between IGF2BP3 and CD44 mRNA by forming a ternary complex, which in turn facilitates IGF2BP3 to promote the levels of CD44, ultimately leading to cell malignant phenotype in the GC (67).

Zhang et al. recruited 96 early gastric cancer (EGC) patients after endoscopic submucosal dissection (ESD) treatment (68). And the patients were divided into two groups with high or low circFNDC3B expression. In high expression group, miR-942 and miR-510 were down-expressed and CD44 and CDH1 were up-expressed. And higher expression of CD44 and CDH1 was identified to be strongly associated higher recurrence rate. Interestingly, they found H. pylori infection could promote circFNDC3B expression, which also resulted in up-expression of CD44 and CDH1 mRNA in rTip-α co-cultured MKN28 cells (68). In conclusion, circFNDC3B had particularly valuable significance in the diagnosis of GC.

### Esophageal cancer

Esophageal cancer (ESCC) is one of the most common malignancies in developing countries, with half of them occurring in China (71–73). Based on pathological classification, esophageal carcinoma is mainly divided into squamous cell carcinoma, adenocarcinoma and other rare types (74). Several researches had been conducted to investigate the role of circFNDC3B in the malignant progression of esophageal cancer by Luo et al., Tang et al., and Wang et al. (75–77).

The experimental results of Luo et al. showed that circFNDC3B was involved in promoting the proliferation, migration and invasion and inhibiting the apoptosis of ESCCcells (75). circFNDC3B was proved stable in ESCC through RNase R digestion. Moreover, the expression level of circFNDC3B was detected in 23 pairs of ESCC and adjacent tissues, and the results showed that circFNDC3B was significantly upregulated in cancer tissues. Silencing circFNDC3B could observably inhibit the development of ESCC in many aspects. For instance, knockdown of circFNDC3B could inhibit tumor proliferation, migration and invasion. In the meantime, the apoptosis rate of tumor cells increased, reflecting the effect of circFNDC3B in inhibiting the apoptosis of esophageal cancer. The focus of this study was the expression and function of circFNDC3B in ESCC cells, but the potential molecular mechanism had not been further studied, which was regrettable.

Tang et al. found circFNDC3B was upregulated in ESCC tissues and ESCC-derived exosomes (76). The knockout expression of circFNDC3B in exosomes significantly inhibited the growth of co-incubated tumor cells. Through dual-luciferase reporter assay, RNA pull-down assay and RNA immunoprecipitation (RIP) assay, miR-490-5p/TXNRD1 axis was identified as the biological function pathway of circFNDC3B, implying circFNDC3B could be a potential indicator for diagnosing ESCC.

Wang et al. also proved circFNDC3B was highly expressed in ESCC (77). And through the experiments *in vitro* and *in vivo*, they confirmed the function of circFNDC3B in ESCC. Unlike the molecular mechanisms discovered by Tang et al., Wang et al. identified miR-214-3p/CDC25A axis was a possible way in which circFNDC3B functions, which indicating circFNDC3B might be the spongy site of multiple miRNAs.

### Colorectal cancer

Colorectal cancer (CRC), which are also called bowel cancer, rectal cancer or colon cancer (CC), is acknowledged as one of the major cancers, which is accounting for approximately one-tenth of all cancer cases, making it one of the three cancers with the highest morbidity and mortality (78, 79). In despite of the significant advances made in clinical strategies, the survival of CRC patients is not satisfactory (80). Therefore, novel therapeutic targets for the diagnosis and prognosis of CRC are urgently needed. CircFNDC3B was an originally identified promising biomarker that regulated CRC development (30, 81, 82).

Pan et al. found that, compared with normal counterparts, circFNDC3B was lower expressed in CC tissues, and its low expression level was positively correlated with lymphatic metastasis (81). Meanwhile, Kaplan-Meier survival curve demonstrated that patients with lower expression level of circFNDC3B had greatly shorter OS than those with higher expression level. Experiments in CC cell lines indicated that overexpression of circFNDC3B repressed the proliferation, migration and invasion ability of tumor cells, while silencing the expression of circFNDC3B could produce opposite results. By matching circFNDC3B with circRNADb, Pan et al. found that circFNDC3B contains an open reading frame that encodes 218 amino acids (circFNDC3B-218aa). The identification of circFNDC3B-218aa was performed using LC-MS/MS. Further experiments showed that circFNDC3B-218aa, rather than circFNDC3B, regulated the malignant progression of CC. Furthermore, circFNDC3B-218aa was proved to repress the cancer progression and EMT by moderating the suppressive effect of Snail on FBP1 *in vivo* and *in vitro*. Considering that FBP1, as a gluconeogenic regulatory enzyme, has been shown to play an important role in the impairment of aggressive phenotypes in various cancers through the metabolic switch from glycolysis to oxidative phosphorylation (OXPHOS), Pan et al. investigated whether circFNDC3B-218aa inhibits EMT by participating in Warburg effect inhibition through FBP1. And the results confirmed that circFNDC3B-218aa boosted metabolic reprogramming in glycolysis and oxidative phosphorylation by inhibiting Snail-FBP1 signaling axis, and then inhibited EMT progression.

By analyzing data from exoRBase, Zeng et al. identified 387 circRNAs that were differentially expressed in plasma exosomes from healthy individuals (n = 32) and CRC patients (n = 12) (30). GO analysis confirmed that circFNDC3B was associated with tumor metastasis. Meanwhile, Zeng et al. collected exosomes from cultures of CRC cell lines and performed circRNAs identification, and found that circFNDC3B expression was down-regulated in CRC cell lines. In terms of the mechanism of action of circFNDC3B, Zeng et al. found that miR-937-5p expression was increased in both CRC cell lines and tumor tissues of CRC patients, so they speculated that circFNDC3B could inhibit the progression of CRC by sponge interaction with miR-937-5p. The expression of miR-937-5p was detected by up-regulating or silencing the expression of circFNDC3B, and the dual luciferase assay and RIP assay confirmed that circFNDC3B and miR-937-5p could be directly bound and negatively correlated. In terms of the function of circFNDC3B, overexpression of circFNDC3B significantly inhibited cell proliferation, migration, invasion, colony formation, angiogenic properties and EMT, and silencing circFNDC3B produced opposite results. Significantly, the effect of circFNDC3B on CRC was partially reversed by miR-937-5p. Using TargetScan tool and dual luciferase assay, Zeng et al. found that miR-937-5p can bind to TIMP3, and TIMP3 can reverse the effects produced by miR-937-5p on CRC proliferation, migration, invasion, colony formation, angiogenesis and EMT. Meanwhile, Zeng et al. also confirmed that circFNDC3B could regulate CRC tumor progression, angiogenesis and liver metastasis through miR-937-5p/TIMP3 axis *in vivo*.

Zeng et al. also found circFNDC3B was down-expressed in CRC tissues and low expression was related to poor OS (82). Experiments *in vitro* confirmed circFNDC3B could modulate CRC stemness and metastasis. Mechanistically, they confirmed N6-methyladenosine (m^6^A)-modified circFNDC3B was regulated by YTHDC1, and circFNDC3B promoted RNF41 expression *via* integrating FXR2. And circFNDC3B facilitated ASB6 degradation *via* RNF41-mediated ubiquitination. The results not only deepened the understanding of m^6^A-modified ncRNAs, but also provided new candidates to identify targeted therapies for CRC.

### Renal carcinoma

Renal carcinoma (RC) is another common urologic tumor (83). Although great progress has been made in its diagnosis and treatment, the overall prognosis is not satisfactory (84, 85). Curcumin, the active ingredient in turmeric root, plays an important role in cardiovascular and neurological diseases, as well as some cancers (86). Recently, Xue et al. gave a new explanation for the therapeutic effect of curcumin in RC and related molecular mechanism (87).

Xue et al. found that circFNDC3B expression was elevated in RC tissues (87). After treatment with different doses of curcumin, it was found that the proliferation ability of RC cells was negatively correlated with the concentration of curcumin, while the apoptosis ability was positively correlated with the concentration of curcumin. After curcumin treatment, the expression of circFNDC3B in RC cells was significantly decreased, and the up-regulation of circFNDC3B significantly alleviated the inhibition of proliferation and clone formation of RC cells by curcumin, and reversed the cell apoptosis induced by curcumin. MiR-138-5p, which was downregulated in RC tissues, was the target of circFNDC3B screened by starBase. Silencing miR-138-5p can significantly attenuate the proliferation inhibition and pro-apoptosis effects of curcumin on RC cells. Similarly, IGF2 had been identified as the target of miR-138-5p and curcumin could reduce the expression of IGF2 by regulating the circFNDC3B expression. In conclusion, curcumin inhibits RC tumorigenesis through repressing cell proliferation and inducing cell apoptosis through circFNDC3B/miR-138-5p/IGF2 network.

### Oral squamous cell carcinoma

Oral squamous cell carcinoma (OSCC), which originate from oropharynx and mouth, is one of the most prevalent head and neck malignancies (88). The incidence of OSCC is increasing year by year due to poor dietary habits and related infectious diseases, and the satisfactory treatment strategies have not been developed, which makes the 5-year survival rate of OSCC low (89, 90). Therefore, clarifying the molecular mechanism of OSCC is of great significance for prevention and treatment of OSCC. One research proved that circFNDC3B reduced ferroptosis of OSCC cells and promoted the progression of OSCC by regulating miR-520d-5p/SLC7A11 axis (91). And another research found circFNDC3B modulated the development of oral tongue squamous cell carcinoma (OTSCC) through miR1322/MED1 axis (92).

Yang et al. identified that the expression of circFNDC3B was negative related with the prognosis of OSCC patients (91). Overexpression of circFNDC3B could promote the proliferation and inhibit apoptosis of OSCC cells *in vitro*, while silencing circFNDC3B could cause opposite results. Interestingly, silencing circFNDC3B inhibited the expression of GPX4 and SLC7A11, the negative regulators of ferroptosis, in OSCC cells. Using ENCORI Online Database, Yang et al. found that miR-520d-5p could be negative associated with circFNDC3B and SLC7A11, which was verified in clinical tissue samples. In addition, erastin, an inducer of ferroptosis, further enhanced ferroptosis in circFNDC3B silenced OSCC cells, and this effect was reversed by SLC7A11 overexpression, which was further demonstrated that circFNDC3B could inhibit ferroptosis in OSCC cells though inducing SLC7A11 expression. Using the same protocol, Yang et al. also demonstrated the role of miR-520d-5p in ferroptosis and the progression in OSCC cells. Taken together, circFNDC3B reduced ferroptosis and promoted the progression of OSCC by modulating miR-520d-5p/SLC7A11 axis.

In addition, Chen et al. found that the expression level of circFNDC3B in OTSCC tissues and cell lines was significantly higher than that in the control group (92). Knockdown of circFNDC3B could inhibit the proliferation, migration and invasion of OSCC cells, and this effect could be reversed by overexpression miR-1322. As the target of miR-1322, MED1 was proved to be upregulated by overexpressed circFNDC3B and play a role in promoting the progression of OTSCC. These two studies confirmed that circFNDC3B played a cancer-promoting role in OSCC, although the mechanisms of action were different.

## The roles of circFNDC3B in other diseases

### Myocardial infarction

Cardiovascular disease is another disease that threatens human life besides tumor, among which myocardial infarction (MI) accounts for an important share (93, 94). At present, the treatment of MI pays more attention to medication, but the effect is still not satisfactory (95, 96). In order to better understand the molecular mechanisms of cardiac repair and cardiac function remodeling after MI, researchers have invested a lot of efforts (97–99).

Garikipati et al. first constructed a mouse myocardial infarction model (100). RNA extracted from mouse hearts three days after MI was compared with the sham operation group, and it was found that circFNDC3B was significantly down-regulated in the heart after MI, and the expression of circFNDC3B continued to decrease within 6 weeks after MI. After up-regulation of circFNDC3B in mouse cardiac endothelial cells (MCECs), it was found that overexpression of circFNDC3B could promote the expression of vascular endothelial growth factor-A (VEGF-A), an angiogenic gene, and inhibit the apoptosis of MCECs. In addition, circFNDC3B viral particles was injected into the myocardium of post-MI model mice. Echocardiography showed that circFNDC3B could significantly relieve left ventricular (LV) dysfunction after MI, promote the generation of myocardial neovascularization and reduce the infarct area. It is worth noting that circFNDC3B has potential sponge binding miRNAs, such as miR-93-3p, miR-298-5p and miR-412-3p, but the experimental results showed that the sponge effect of circFNDC3B was not significant in cardiac repair after MI either *in vivo* or *in vitro*. However, Garikipati et al. found that circFNDC3B can bind protein fused in sarcoma (FUS) and promote expression of VEGF by down-regulating FUS expression, which provides an attractive therapeutic option for MI patients.

### Abdominal aortic aneurysm

Abdominal aortic aneurysm (AAA) is a degenerative vascular disease closely related to the dysfunction of vascular smooth muscle cells (VSMCs) (101, 102). At present, the potential mechanisms of VSMCs dysfunction have been reported in relevant studies, aiming to provide new strategies for the prevention and treatment of AAA (103–105).

Liu et al. demonstrated that circFNDC3B expression was elevated in aortic tissue of AAA patients (106). They further isolated and cultured primary VSMCs from AAA patients and found that the expression of circFNDC3B was higher in them and angiotensin II (Ang-II) induced circFNDC3B expression in a dose-dependent manner. In terms of biological effects of circFNDC3B, silencing circFNDC3B significantly reduced pro-inflammatory cytokine IL-6 and TNF-α production induced by Ang-II, and also alleviated Ang-II-mediated inhibition of cell proliferation in VSMCs. In addition, silencing circFNDC3B significantly suppressed Ang-II-mediated inhibition of superoxide dismutase level and increased malondialdehyde content in VSMCs. In terms of the mechanism of circFNDC3B in VSMCs, miR-143-3p had been shown to directly bind and antagonize circFNDC3B, while ADAM10 was a downstream target of mirR-143-3p. Thus, circFNDC3B was illustrated to regulate cell proliferation, apoptosis, inflammation and oxidative stress through miR-143-3p/ADAM10 axis to modulate Ang-II-induced cell damage (**Table 1**).

**Table 1:** Functional roles of circFNDC3B in different cancers and other diseases.

First author, year	Disease	Expression	Mechanism	Functional roles	Reference
Liu et al., 2018	Bladder cancer	Down-regulated	miR-1178-3p/G3BP2/SRC/FAK axis	Proliferation and invasion	(62)
Luo et al., 2018	Esophageal cancer	Up-regulated	Unknown	Proliferation, invasion, migration and apoptosis	(75)
Tang et al., 2022	Esophageal cancer	Up-regulated	miR-490-5p/TXNRD1 axis	Colony formation, proliferation, migration, invasion, glycolysis	(76)
Wang et al., 2022	Esophageal cancer	Up-regulated	miR-214-3p/CDC25A axis	Cell growth, migration and invasion	(77)
Hong et al., 2019	Gastric cancer	Up-regulated	circFNDC3B‐IGF2BP3‐CD44 mRNA ternary complex	Migration, invasion and EMT	(67)
Zhang et al., 2022	Gastric cancer	Up-regulated	miR-942/CD44 and miR-510/CDH1 axes	Recurrence-free rate	(68)
Pan et al., 2020	Colon cancer	Down-regulated	Encoding circFNDC3B-218aa	Proliferation, migration, invasion and EMT	(81)
Zeng et al., 2020	Colorectal cancer	Down-regulated	miR-937-5p/TIMP3 pathway	Proliferation, migration, invasion, angiogenesis and EMT	(30)
Zeng et al., 2022	Colorectal cancer	Down-regulated	YTHDC1/m^6^A-circFNDC3B/FXR2/RNF41/ASB6 axis	Spheres formation, migration, invasion	(82)
Xue et al., 2021	Renal carcinoma	Up-regulated	miR-138-5p/IGF2 network	Proliferation and apoptosis	(87)
Yang et al., 2021	Oral squamous cell carcinoma	Up-regulated	miR-520d-5p/SLC7A11 axis	Proliferation, viability and apoptosis	(91)
Chen et al., 2022	Oral tongue squamous cell carcinoma	Up-regulated	miR-1322/MED1 axis	Proliferation, migration, and invasion	(92)
Garikipati et al., 2019	Myocardial infarction	Down-regulated	FUS/VEGF-A signaling network	Cardiomyocyte apoptosis, neovascularization, infarct size, and post-MI cardiac function and integrity	(100)
Liu et al., 2021	Abdominal aortic aneurysm	Up-regulated	miR-143-3p/ADAM10 axis	Proliferation, viability, inflammation and apoptosis	(106)

## Conclusions and future perspectives

In recent years, with the rapid development of high throughput sequencing technology and bioinformatics, the mysterious veil of circRNAs is gradually being revealed (42, 107). From originally being regarded as by-products of selective splicing to being used as biomarkers of disease now, the biological function of circRNAs has attracted more attention. Recent studies have shown that many of abnormal expressions of circRNAs are closely related to the occurrence and development of diseases. CircRNAs are gradually becoming vital biomarkers and targets for disease diagnosis and treatment.

At present, researchers are eager to explore more circRNAs related to tumorigenesis and development, but rarely focus on a single meaningful circRNA to elaborate its role in different tumors. Although this provides more options for potential biological markers for cancer, it also creates difficulties in determining the application of a specific molecule. CircHIPK3 and circFOXO3 are two representative molecules that have been studied in a variety of tumors and other diseases (108, 109). Unfortunately, the mechanism of action of both is still limited to miRNA sponge. In this review, we first systematically reviewed and discussed the production process and related functions of circular RNA. At the same time, we focused on circFNDC3B and elaborated the production process, basic characteristics, multiple function mode and role in disease progression of circFNDC3B.

In combination with the above studies, we found that circFNDC3B can act as miRNA sponges and form ceRNA network and circRNA-miRNA-mRNA axis to regulate related signal pathways. Since Hansen et al. first proposed that circRNAs can play a role as miRNA sponge in 2013, miRNA sponge has always been a research hotspot of circRNAs (110). CircRNAs, binding to miRNAs through multiple miRNA response elements (MREs), inhibit miRNA activity and thereby weaken the inhibition of miRNA on their target genes (111–113). The mechanism of miRNA sponge is very suitable for clinical research due to the rapid development of bioinformatics prediction websites and the fact that miRNA sponge can be easily verified by RNA pulldown experiments. However, because of the lack of enough miRNA binding sites in circRNAs, and the abundance of most circRNAs is far lower than that of miRNAs, more and more scholars have questioned the efficiency of circRNA binding to miRNAs (114, 115). In addition, circFNDC3B can also form ternary complex by binding proteins or encode circFNDC3B-218aa protein to play a biological role. RNA binding proteins is another mode of function of circRNAs (116). CircRNAs can directly or indirectly regulate the transcription or translation process of target proteins by interacting with proteins through protein recruitment, protein scaffold and other forms (117). In addition, RBP can also mediate proteins ubiquitination and phosphorylation degradation (35, 116). Like miRNA sponge, the abundance of circRNAs should also be considered when clarifying the potential protein binding mechanism (118). It is worth noting that the binding of circRNAs to proteins may depend not only on the nucleotide sequence, but also on the secondary or tertiary structure between circRNAs and proteins (119). CircRNAs can also play a role by coding proteins or peptides (116, 118). N^6^-methyladenosine (m^6^A) modification can promote the translation of circRNAs into small peptides and this process can be carried out in a way that does not rely on the 5’ cap (120). M^6^A modification is an epigenetic modification that widely exists in circRNAs, and plays an important role in regulating gene expression, splicing, RNA editing, RNA stability and controlling mRNA longevity and degradation (121, 122). However, most circRNAs originate from the back splicing of exons, which may cause the corresponding mRNA level to decrease. Correspondingly, proteins coding by circRNAs are usually “abridged version” compared with linear proteins (123, 124). Whether the proteins originated from circRNAs have similar functions to the corresponding linear RNA encoded proteins is still uncertain (123). Interestingly, only one naturally occurring circRNA has been found to encode protein in eukaryotic cells, namely hepatitis D virus (HDV) (125). Ribosome footprint detection has also proved that there is no translatable circRNAs in osteosarcoma (21, 126). Therefore, the protein encoding process of circRNAs in eukaryotes still needs further exploration.

It is worth noting that the way circRNAs function is not independent of each other, so circFNDC3B is likely to co-regulate the occurrence of diseases through multiple modes of action at the same time. This also suggests that the functions of circRNAs are complex and the research of circRNAs needs to be more comprehensive. In addition, the current studies explored more about the dysregulated circRNAs in cell lines and tissues rather than in peripheral blood. Thus, it is significant to investigate the specific functions of circRNAs in different specimen sources.

CircFNDC3B is a molecule that has been found to regulate progression in a variety of tumors and other diseases. It is up-regulated in esophageal cancer, gastric cancer, renal cancer, oral squamous cell carcinoma and thyroid cancer, but is down-regulated in bladder cancer and colorectal cancer. The expression of circFNDC3B is closely related to a variety of clinical characteristics (such as TNM stage, histological grade, lymphatic metastasis, etc.). Moreover, it is also closely related to non-tumor diseases such as myocardial infarction and abdominal aortic aneurysm. CircFNDC3B is dysregulated in this series of diseases and participates in inflammation, cell proliferation, invasion, migration, apoptosis and other biological phenomena. Compared with linear FNDC3B, circFNDC3B has the characteristics of stability, conservatism, specificity, universality and so on (42). Therefore, circFNDC3B is expected to become a specific biomarker to predict the occurrence of cancer or other diseases. At the same time, considering the diverse mechanisms of action of circFNDC3B, we can utilize its unique ability of sponge miRNAs and proteins to make it a potential therapeutic drug carrier, and can also use the proteins encoded by circFNDC3B to restore uncontrolled cell proliferation or induce apoptosis, making circFNDC3B an important molecule for clinical treatment.

However, we still find that the current researches of circRNAs have some limitations. Most current circRNAs researches are typically based on the data from RNA-seq, and the experimental designs and library preparations are in line with these results (127, 128). Therefore, it may lead to a probable bias in the identification and prediction analysis of circRNA populations. The heterogeneity of different circRNA recognition algorithms also leads to great differences in their sensitivity and specificity to circRNA, so that low-abundance but meaningful circRNAs are ignored (129).

Based on this review, we focus on the roles and related mechanisms of circFNDC3B in various diseases, aiming to identify a potential star molecule with comprehensive functional approaches that could provide a valuable option of targeting molecules for cancer diagnosis and treatment.

## Author contributions

KS and HY were for the design of the review, and the writing of manuscript. PZ and YS was for the collection of related papers, and the writing of tables and figures. JM and QX revised the manuscript. All authors approved the final manuscript. All authors contributed to the article and approved the submitted version.
